# Associations between COVID-19 lockdown and post-lockdown on the mental health of pregnant women, postpartum women and their partners from the Queensland family cohort prospective study

**DOI:** 10.1186/s12884-022-04795-9

**Published:** 2022-06-04

**Authors:** Vicki L. Clifton, Sailesh Kumar, Danielle Borg, Kym M. Rae, Roslyn N. Boyd, Koa Whittingham, Karen M. Moritz, Hannah E. Carter, Steven M. McPhail, Brenda Gannon, Robert Ware, Barnaby J. W. Dixson, Samudragupta Bora, Cameron Hurst

**Affiliations:** 1 Mater Research Institute, The University of Queensland, Raymond Terrace, Level 3, Aubigny Place, South Brisbane, QLD 4101 Australia; 2 Faculty of Medicine, The University of Queensland, Brisbane, Australia; 3 Faculty of Medicine, Queensland Cerebral Palsy and Rehabilitation Research Centre, Child Health Research Centre, The University of Queensland, Brisbane, Australia; 4 School of Biomedical Sciences, the Child Health Research Centre, The University of Queensland, Brisbane, Australia; 5 Australian Centre for Health Services Innovation and Centre for Healthcare Transformation, School of Public Health and Social Work, Queensland University of Technology, Brisbane, Australia; 6 Clinical Informatics Directorate, Metro South Health, Brisbane, Australia; 7 Centre for the Business and Economics of Health and School of Economics, The University of Queensland, Brisbane, Australia; 8 Menzies Health Institute Queensland, Griffith University, Brisbane, Australia; 9 School of Health and Behavioural Sciences, University of the Sunshine Coast, Queensland, Australia; 10 School of Psychology, The University of Queensland, Queensland, Australia; 11 Molly Wardaguga Research Centre, Charles Darwin University, Queensland, Australia

**Keywords:** Pregnancy, Postpartum, Mental health, Anxiety, Depression, Stress, COVID-19

## Abstract

**Background:**

There are very few developed countries where physical isolation and low community transmission has been reported for COVID-19 but this has been the experience of Australia. The impact of physical isolation combined with low disease transmission on the mental health of pregnant women is currently unknown and there have been no studies examining the psychological experience for partners of pregnant women during lockdown. The aim of the current study was to examine the impact of the first COVID-19 lockdown in March 2020 and post lockdown from August 2020 on the mental health of pregnant women or postpartum women and their partners.

**Methods:**

Pregnant women and their partners were prospectively recruited to the study before 24 weeks gestation and completed various questionnaires related to mental health and general wellbeing at 24 weeks gestation and then again at 6 weeks postpartum. The Depression, Anxiety and Stress Scale (DASS-21) and the Edinburgh Postnatal Depression Scale (EPDS) were used as outcome measures for the assessment of mental health in women and DASS-21 was administered to their partners. This analysis encompasses 3 time points where families were recruited; before the pandemic (Aug 2018-Feb 2020), during lockdown (Mar-Aug 2020) and after the first lockdown was over (Sept-Dec 2020).

**Results:**

There was no significant effect of COVID-19 lockdown and post lockdown on depression or postnatal depression in women when compared to a pre-COVID-19 subgroup. The odds of pregnant women or postpartum women experiencing severe anxiety was more than halved in women during lockdown relative to women in the pre-COVID-19 period (OR = 0.47; 95%CI: 0.27–0.81; *P* = 0.006). Following lockdown severe anxiety was comparable to the pre-COVID-19 women. Lockdown did not have any substantial effects on stress scores for pregnant and postpartum women. However, a substantial decrease of over 70% in the odds of severe stress was observed post-lockdown relative to pre-COVID-19 levels. Partner’s depression, anxiety and stress did not change significantly with lockdown or post lockdown.

**Conclusion:**

A reproductive age population appear to be able to manage the impact of lockdown and the pandemic with some benefits related to reduced anxiety.

**Supplementary Information:**

The online version contains supplementary material available at 10.1186/s12884-022-04795-9.

## Introduction

In December 2019, an outbreak of pneumonia with unknown origins was identified in Wuhan, China and subsequently identified as a virus known as severe acute respiratory syndrome coronavirus 2 (SARS-CoV-2) resulting in the respiratory disease known as coronavirus disease of 2019 (COVID19). This virus has rapidly spread contributing to 4.2 million deaths globally. In Australia there have been > 42,000 confirmed cases identified with 971 deaths (www.who.int/covid-19).

To date the main approach to reduce the numbers of infections has been to lockdown entire cities or states and enforce physical isolation. Preliminary evidence from Australia indicates that the COVID-19 response and lockdown has resulted in declines in employment and income, changes in household structure, and increases in social isolation, psychological distress and financial stress [1]. Previous studies during the 2009 H1N1 influenza outbreak have shown that school closures led to missed work days for parents and carers and negative financial consequences for many families with school age children [2].

At the time of COVID-19 pandemic onset the implications of physical isolation and community lockdowns for the mental health of women during a pregnancy and postpartum were unknown. A study in China reported a 3% increase in depressive symptoms in the pregnant women with onset of SARS-CoV-2 pandemic lockdown [3]. Similarly, in Canada pregnant women reported increased depressive and anxiety symptoms during COVID19 pandemic lockdown especially if they had a previous mental health disorder or were from a socially disadvantaged community [4]. In the Netherlands a prospective cohort of pregnant women reported increased stress but no change in depression or postnatal depressive symptoms with COVID-19 lockdown [5]. From a comparison of three countries, it was highlighted a variety of factors negatively influenced maternal health during a COVID-19 lockdown including advanced maternal age, poor physical health, higher maternal education and unemployment. In contrast, having more than one child, being married, and having grandparental support for mothers were important protective factors for lowering the risk for mental health symptoms [6]. Moreover, high socioeconomic status (SES) (mother’s high education, high family income) and poor physical health were related to high levels of mental health symptoms among Chinese mothers during lockdown [6]. There are very few developed countries where enforced physical isolation and low community transmission has been reported but this has been the experience of Australia and New Zealand. The impact of physical isolation combined with low disease transmission on mental health of pregnant women is currently unknown. Furthermore, there have been no studies examining the psychological experience of fathers or partners of pregnant women during COVID-19 lockdown.

The aim of the current study was to examine the impact of the first COVID-19 lockdown in March 2020 and post lockdown from August 2020 on the mental health of pregnant women and their partners in Brisbane, Australia. Our study accounted for the effect of income, education, couples’ relationship satisfaction, quality of life, numbers of children in the household and social support as drivers of altered mental health symptoms.

### Methodology

The data for this sub-study was collected as part of an ongoing life-course pregnancy study known as the Queensland Family Cohort being conducted at the Mater Mother’s Hospital in Brisbane, Australia. The Queensland Family Cohort was commenced in August 2018 and its protocol has been published in detail [7]. Pregnant women and their partners (any gender or sex), and single women were prospectively recruited to the study before 24 weeks gestation at Mater Mothers Hospital (MMH) in Brisbane, Queensland, Australia (*n* = 454 families). The MMH is a tertiary level obstetric hospital that provides both a private and public obstetric health service. Families were recruited from both the public and private health services via an initial opt-in for research check box on their booking form. Potential participants were then contacted by text and then phone about the study, provided information and consent forms via email and then contacted by phone for enrolment after returning the consent form. This research was approved by Mater Human Research Ethics (HREC/16/MHS/113) and Governance Committees and The University of Queensland Human Research Ethics Committee (2017/HE001443). All methods were carried out in accordance with relevant guidelines and regulations of Mater Research, the University of Queensland and in line with Australian guidelines for ethical conduct of research. Informed consent was obtained from all subjects or their legal guardian to participate in the study.

#### Measures and scoring

Pregnant women completed various questionnaires related to mental health and general wellbeing at 24, 28 and 36 weeks gestation and then again at 6 weeks postpartum. Partners (any gender) completed the same questionnaires at one time point only when their pregnant partner was at 24 weeks gestation. Pregnant women answered the Depression, Anxiety and Stress Scale (DASS-21) [8] at 24 weeks gestation and again at 6 weeks postpartum as well as the Edinburgh Postnatal Depression Scale (EPDS) [9]. Their partner completed a DASS-21 survey at the 24 week visit. Other questionnaires included in this analysis are the Assessment of Quality of Life 6D (AQoL) [10], Couples Satisfaction Index (CSI) [11], Multidimensional Scale of Perceived Social Support (MSPSS) [12] and Social Readjustment Rating Scale (SRRS) [13]. Data on occupation, education, weekly income after tax, gravidity, parity, pre-existing chronic disease and pregnancy complications were also recorded. Pre-pregnancy and weight at enrolment (22 weeks) were self-reported variables. Before the pandemic, weight and height were measured at face to face visits at 24 weeks and 6 weeks post-partum. During and after lockdown, weight and height were self-reported by families in a telehealth appointment with a research midwife. Body mass index (BMI) was calculated for each participant [weight (kg)/height^2^ (m^2^)] and were classified as underweight (BMI ≤ 18.5 kg/m^2^), normal weight (BMI 18.5–24.9 kg/m^2^), overweight (BMI 25.0–29.9 kg/m^2^) or obese (≥ 30.0 kg/m^2^).

##### Assessment of Quality of Life Questionnaire (AQoL-6D)

This instrument is a multi-dimensional measure of health-related quality of life [10]. It comprises self-report rating scales for 20-items that assesses dimensions of independent living, mental health, coping, relationships, pain and senses. Each item is scored from 1–5 (Never – nearly all the time). The score for each dimension is derived by adding the unweighted scores for each question within the dimension. This tool can be used as a health profile assessment or to derive multi-attribute utility through the application of societal preference weights to questionnaire responses [10].

##### Couples Satisfaction Index (CSI)

This is a self-reported 4-item scale of relationship satisfaction. Each item uses a varying response scale with the lowest descriptive term eg extremely unhappy = 0 and increasing in value with the more positive descriptive terms eg perfect = 6. The number of descriptive terms in each item varies from 6 to 7. The total score is the sum of the participant point values which can range from 0 to 81. Higher scores indicate greater couple satisfaction, with scores below 51.5 suggesting significant dissatisfaction with the relationship [11].

##### Depression Anxiety Stress Scale (DASS-21)

The DASS-21 is a self-report questionnaire with three scales that address depression, anxiety and stress. Each of the 3 scales contains 7 items with scores calculated by summing of the relevant items. Scores were multiplied by 2 to calculate final scores [8].

##### Multidimensional Scale of Perceived Social Support (MSPSS)

This 12-item self-report scale determines perceived social support according to three groups; family, friends, and significant other. Each item on the scale is scored from 1 (very strongly disagree) through to 7 (very strongly agree). Internal reliability of the MSPSS has been demonstrated across ages, gender, and life situation [12]. The total score is calculated by summing the results for all items with a possible range between 12 and 84, with a low score indicative of lower perceived social support [12].

##### Social Readjustment Rating Scale (SRRS)

The SRRS provides a measure of impact of significant life events in the previous 12 months including, divorce, change in financial state, and jail term. Each event has been assigned a value reflective of the relative amount of stress the event can cause in the population studied. Scoring is undertaken by adding up the assigned values of each of the items selected by participants. Scores <  = 150 is good, 150–299 at moderate risk of stress related disorder while >  = 300 places individual at significant risk of stress-related disorder in near future [13].

##### Edinburgh Postnatal Depression Scale (EPDS)

This self-reported 10-item scale has been shown to be valid for identification of maternal depression during both during the antenatal and post-natal periods[9]. Each item contains a series of a short descriptive statement scoring the most negative description with the highest value of 3 and more positive with 0. Scores are tallied so that a score < 8 indicates depression is not likely, 9–11 depression possible, 12–13 fairly high possibility of depression, 14 and higher is considered to be a positive screen for depression and recommends a diagnostic assessment. Additionally, if a positive score is identified for question 10 identifies participants at risk of harm or suicide.

#### Participant groups and data collection

There were 3 groups of families included in the analysis. Group 1 were families recruited before the pandemic and lockdown (2018-Feb 2020) (*n* = 243), group 2 were families recruited during the lockdown period (Mar-Aug 2020) (*n* = 156) and group 3 were families recruited after the first lockdown was over (Sept-Dec 2020) (*n* = 55). There were 365 partners in the study of which 99% identified as male sex. There were 89 women who identified as single. All data for the QFC Study were collected and managed using Research Electronic Data Capture (REDCap) electronic data capture tools hosted at University of Queensland [14, 15]. REDCap is a secure, web-based software platform designed to support data capture for research studies [16, 17].

### Statistical analyses

The EPDS and DASS-21 scales were collapsed into a binary variable (severe, not severe) [18] and univariate and multivariable binary logistic regression models were used to estimate both the unadjusted and adjusted associations of the COVID-19 intervention (Pre-, During- and Post- lockdown periods) along with those of the couple satisfaction index, social support, quality of life, social adjustment, as well as the partners corresponding mental health outcomes (DASS-21). As there are likely to be socio-demographic differences between the participants in the three groups, propensity score based inverse probability of treatment weighting was employed to offset any potential confounding effect of these sociodemographic factors. The propensity score models were adjusted for participant age, parity, body mass index, income, education, smoking, and pre-existing chronic conditions (for both partners and pregnant women), and gestational diabetes and preeclampsia for mothers only.

In the multivariable models, two types of adjustment were considered. The first was propensity score adjustment using sample weighting which was based on both sociodemographic and clinical variables. The second was standard multivariable adjustment where other psychometric indicators are represented directly in the model, allowing explicit evaluation of these individual psychometric covariates on various outcomes, along with their individual confounding effects. In addition, the dataset did contain some missing values and these missing values occurred for both continuous and categorical variables so factor analytic-based imputation method for mixed data types was used to generate an imputed dataset. To gauge the effect of potential complete case (or imputation) bias we generated the available case summary statistics and compared them with the corresponding descriptive statistics from the imputed dataset (see Supplementary Tables S1 and S2). All analyses were conducted using the R statistical package (v4.1.1; R Core team, 2021) [19]. The propensity scores were generated and used to weight observations using the twang R library [20], and imputed values were generated using the missMDA R library [21]. A significance level of 0.05 was used.

## Results

### Participant characteristics

There were 472 families recruited with 18 withdrawals due to loss to follow up. Pregnant women (*n* = 454) in the 3 groups (Group 1: Pre COVID-19, Group 2: During COVID-19 lockdown and Group 3: Post COVID-19 lockdown) were not significantly different in any of the demographic parameters such as age, BMI, education, income or smoking status (Table 1).

**Table 1:**
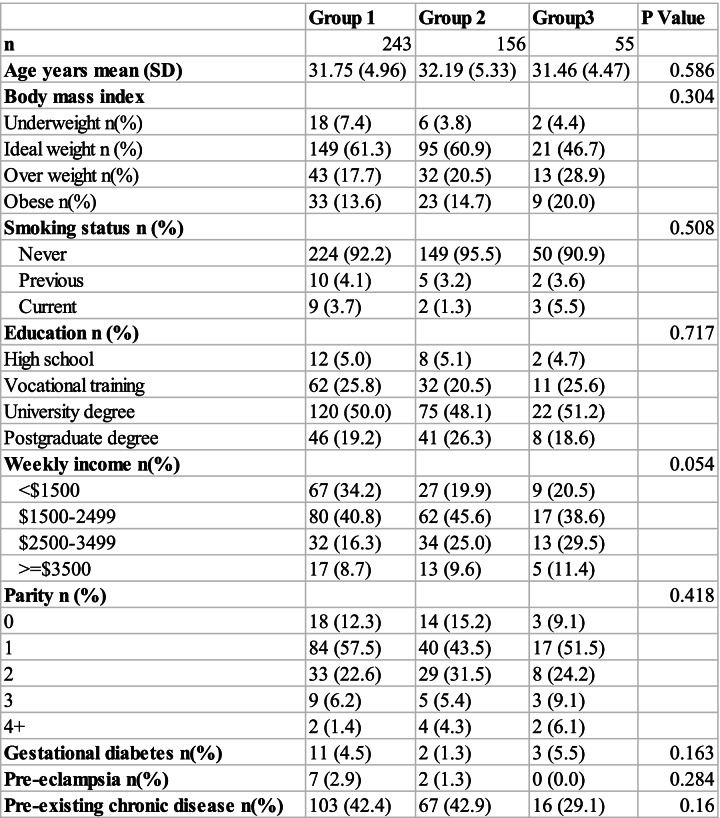
Maternal Demographics

Partners (*n* = 365) were more obese and reported more pre-existing chronic diseases in groups 1 and 2 relative to group 3 (Table 2). In the partners the distribution of the highest level of education both appear to differ substantially across the 3 groups (Table 2). The education differences may be offset by the much higher occurrence of postgraduate education in groups 1 and 2.

**Table 2:**
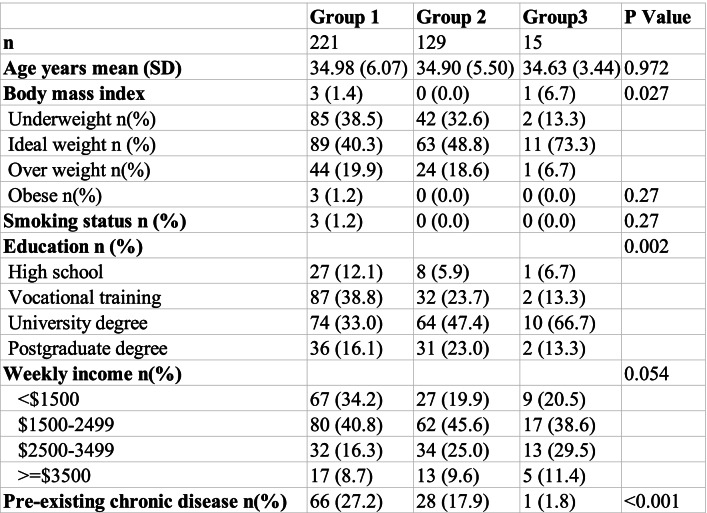
Partner Demographics

The unadjusted scores for each of the questionnaires administered in the study were not significantly different among the 3 groups including data collected on pregnant women at 24 weeks gestation, 6 weeks postpartum and partners (Supplementary Tables S3a, S3b, S3c). The percentage of individuals with severe depression, anxiety and stress was not significantly different between the groups except there were no women with postnatal depression in Group 3 as detected by the EPDS (Supplementary Table S3b).

### Mental health during pregnancy

Maternal depression at 24 weeks gestation was not different between the 3 groups of women with no significant association with lockdown or post lockdown. There were other factors that were highly associated with severe maternal depression. For every unit increase in partner depression score the odds of severe maternal depression was 1.18 times higher (OR = 1.17; 95%CI: 1.06–1.30; *P* < 0.000, Suppl Table S4a).

**Fig. 1:**
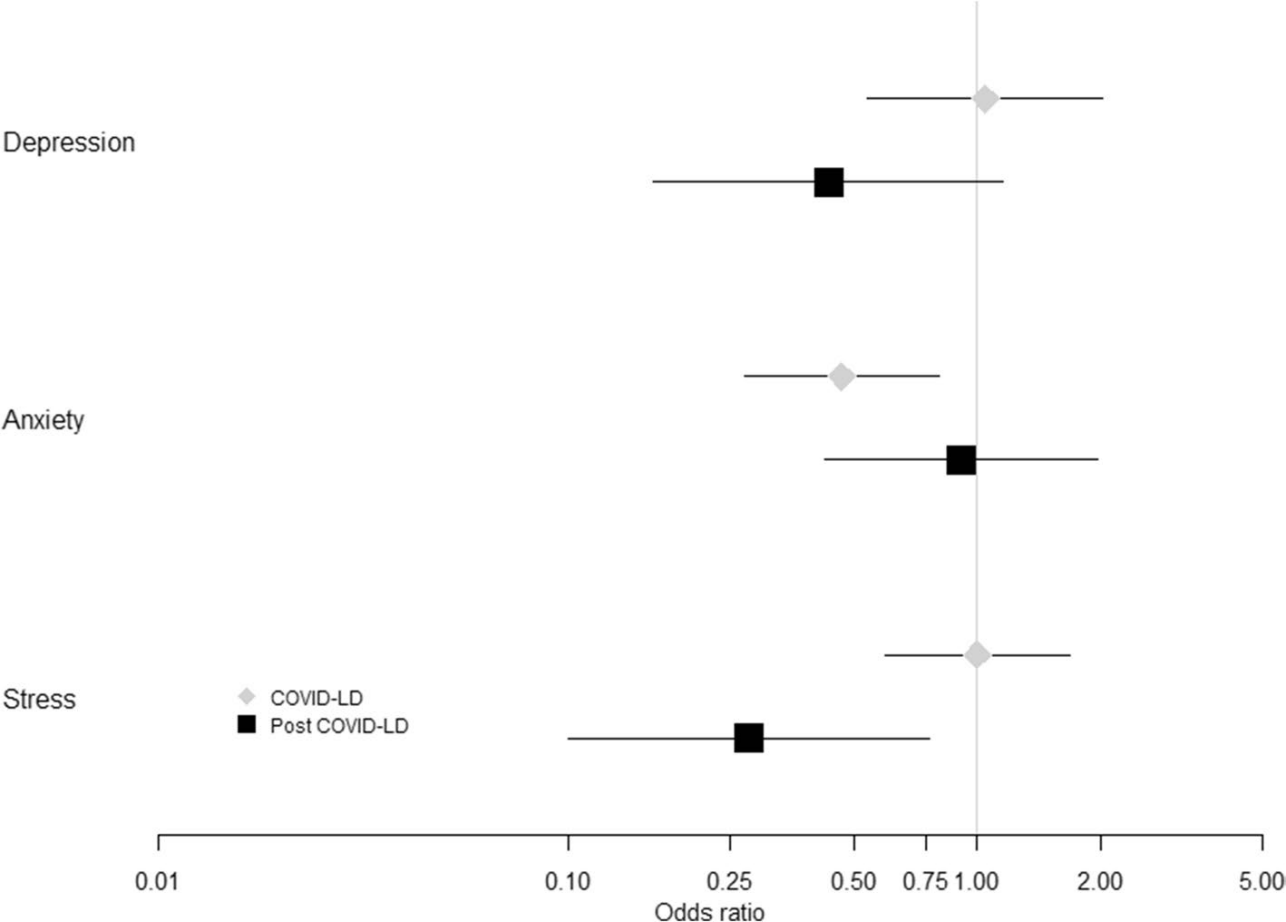
Propensity score adjusted odds of Depression, Anxiety and Stress in pregnant women at 24 weeks gestation during and after COVID19 lockdown relative to pregnant women at 24 weeks gestation before the COVID19 pandemic

Lockdown was shown to have a significant effect on anxiety (Fig. 1). The odds of pregnant women experiencing severe anxiety was more than halved in women during lockdown relative to women in the pre-COVID-19 period (OR = 0.47; 95%CI: 0.27–0.81; *P* = 0.006 Sup Table S4b). Following lockdown severe anxiety was comparable to the pre-COVID-19 women in Group 3 (Fig. 1).

In terms of severe maternal stress, lockdown did not have any substantial effects on stress scores at 24 weeks gestation, as measured by DASS21 (Fig. 1). There was, however, a substantial decrease of over 70% in the odds of severe stress post-lockdown (Group 3) relative to pre-COVID-19 levels (Group 1) (OR = 0.28; 95%CI: 0.10–0.76; *P* = 0.013, Supp Table S4c).

For the most part, the other psychometric measures were not shown to have a substantial effect on any of the DASS21 domains except for AQoL scores. For all three domains (severe depression, severe anxiety and severe stress) a unit increase in AQoL score, representative of a worsening of quality of life, was associated with substantial increase in the odds of a severe status on all three domains (OR_Dep_ = 1.18; OR_Anx_ = 1.27; OR_Str_ = 1.20; All *p* < 0.001, Supp Tables S4a-c).

### Postpartum mental health

**Fig. 2:**
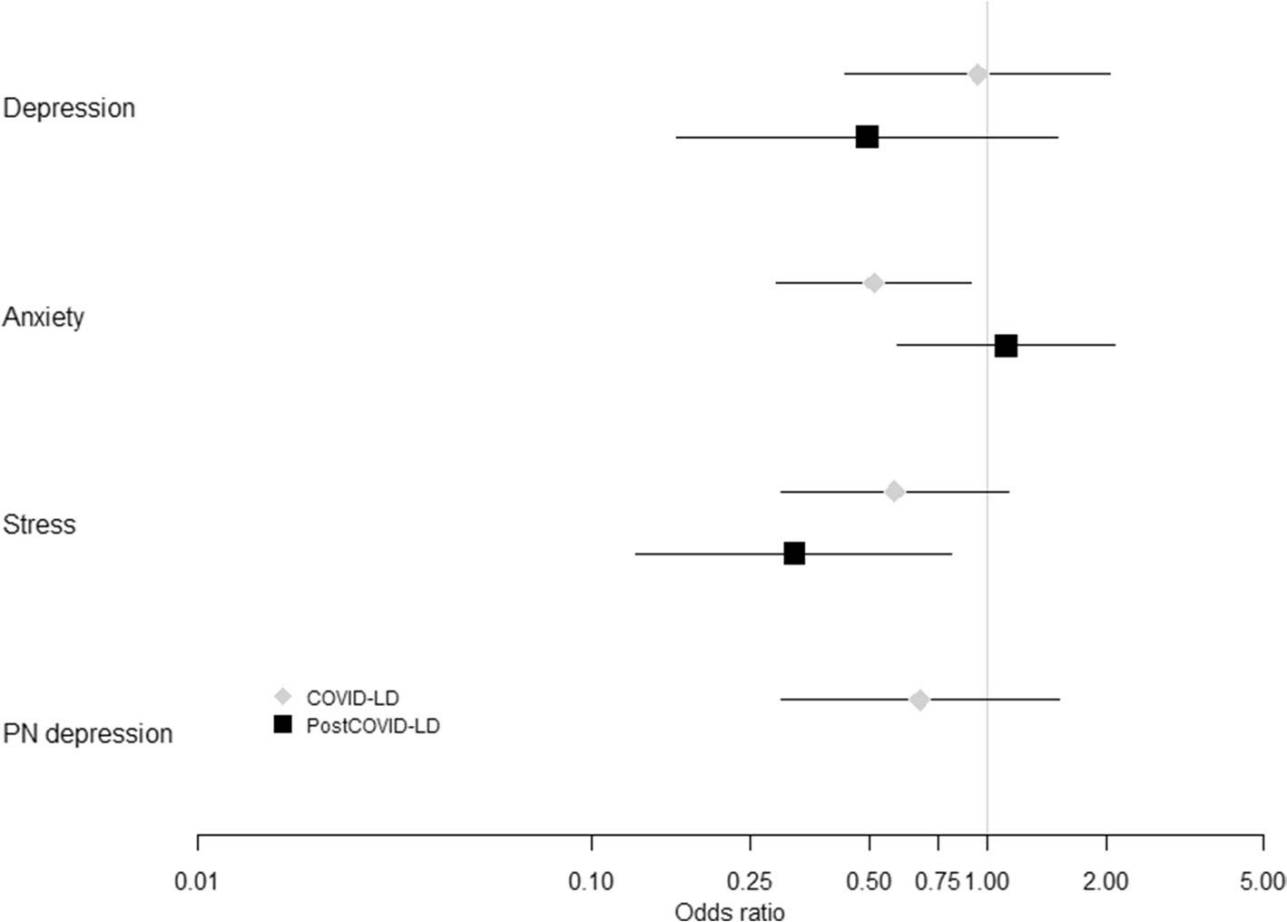
Propensity score adjusted odds of Depression, Anxiety and Stress in women at 6 weeks postpartum during and after COVID19 lockdown relative to women at 6 weeks postpartum before the COVID19 pandemic

Maternal depression scores at 6 weeks postpartum did not change significantly with lockdown or post lockdown relative to women with depression in the pre-COVID-19 period (Fig. 2). Factors that influenced the odds of having severe depression included maternal AQoL and CSI (Supp Table S5a). For every unit increase in maternal AQoL score the odds of severe maternal depression was also 1.3 times higher (OR = 1.29; 95%CI: 1.20–1.38; *P* < 0.001, Supp Table S5a). For every unit increase in maternal CSI score the odds of severe maternal depression decreased by 0.12 (OR = 0.88; 95%CI: 0.81–0.96 *P* = 0.002, Suppl Table S5a).

The odds of women experiencing severe anxiety in the postpartum period during lockdown was 49% lower than postpartum women in the pre-COVID-19 group (Fig. 2, *P* = 0.02). Following lockdown anxiety returned to a level comparable to the pre-COVD women (*P* = 0.74, Fig. 2). Maternal AQol significantly associated with anxiety scores for postpartum women in lockdown and post lockdown (Supp Table S5b). For every unit increase in the maternal AQol score, the odds of severe anxiety were 1.2 times higher (OR = 1.22; 95%CI: 1.15–1.31 *P* < 0.001, Supp Table S5b).

Severe maternal stress scores during the post-partum period did not change significantly with lockdown but were significantly improved post lockdown (Fig. 2, *P* = 0.02) relative to post-partum women in the pre-COVID-19 period. The odds of being severely stressed in the postpartum period after lockdown was reduced by 68.6%. For every unit increase in the maternal AQol score, the odds of severe stress were 1.3 times higher (OR = 1.29; 95%CI: 1.21–1.38 *P* < 0.001, Suppl Table S5c).

The EPDS was also administered to women in the postpartum period with only women in the pre-COVID-19 (Group 1) and lockdown period (Group 2) being identified with postpartum depression using the EPDS (Fig. 2). There are no propensity scores for EPDS reported for women in the post lockdown period (Group 3) (Fig. 2). There was no effect of lockdown on postpartum depression scores relative to the pre-COVID-19 women with postpartum depression (Fig. 2, *P* = 0.39). For every unit increase in the maternal AQol score in the postpartum period, the odds of severe postpartum depression were 1.14 times higher (OR = 1.14; 95%CI: 1.07–1.21, *P* < 0.001, Suppl Table S5d).

### Partners mental health examined during pregnancy

**Fig. 3:**
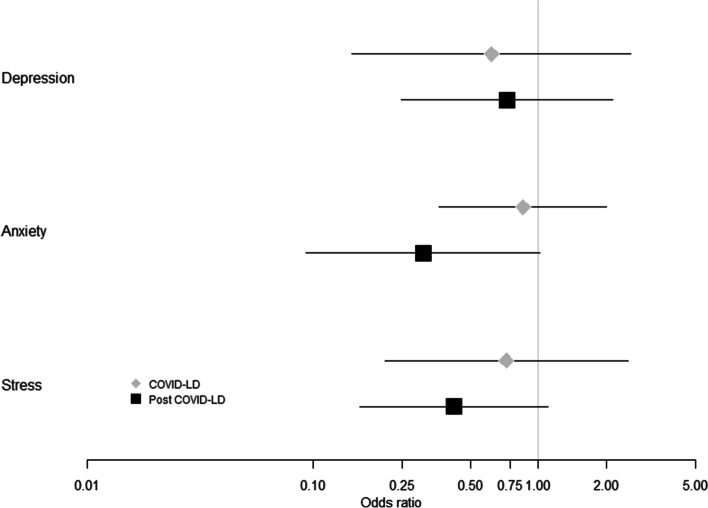
Propensity score adjusted odds of Depression, Anxiety and Stress in partners at 24 weeks gestation during and after COVID19 lockdown relative to partners at 24 weeks gestation before the COVID19 pandemic

Partner’s depression, anxiety and stress did not change significantly with lockdown or post lockdown (Fig. 3, Suppl Table S6a-c). Partner depression, anxiety and stress was significantly associated with their AQol scores. Every unit increase in the paternal AQol score increased the odds of severe depression, anxiety or stress by 1.3 times (P < 0.001, Suppl Table S6a-c).

### Association of AQoL dimensions with outcomes of anxiety, stress, depression, postnatal depression and COVID-1919 lockdown status

It was identified that AQoL was strongly associated with all mental health outcomes in mothers and partners at both 24 weeks and 6 weeks postpartum (data found in Supplementary Figures S1-10). Data was examined to determine if the various AQoL subscale scores which included independent living, mental health, coping, relationships, pain and senses dimensions were associated with lockdown. Neither the overall AQoL scale nor any of the five AQoL subscales differed among any of the groups. We then went on to examine the relationship between the various DASS-21 domains and EPDS with the AQoL subscales to determine if specific AQoL domains might be associated with depression, postnatal depression, stress or anxiety in pregnant women at 24 weeks and 6 weeks postpartum and in the partners. Our analysis revealed that all AQoL subscales had a statistical association with DASS-21 and EPDS measures. In women the relationships, mental health and coping AQoL subscales were especially important during pregnancy and post-partum. For every unit increase in these AQoL scales the odds doubled for poor mental health on the DASS and EPDS scales. For partners, all AQoL domains were significantly associated with depression, anxiety and depression (data provided in supplementary Figures S1-10).

## Discussion

We investigated the mental health outcomes of the parents of an Australian antenatal population during the first wave of the COVID-19 pandemic. This antenatal population were birthing at an inner-city hospital that provides a combination of private and public antenatal services. As a result of this service delivery model and its location, the recruited families were predominantly well educated with a medium to high weekly income. Lockdown of this population was associated with beneficial mental health effects by reducing maternal anxiety at both 24 weeks gestation and 6 weeks postpartum but lockdown was not associated with altered stress, depression or postnatal depression outcomes for women. After lockdown maternal stress was significantly reduced at both 24 weeks gestation and at 6 weeks postpartum but anxiety returned to pre-COVID-19 levels. Partners had no significant change in depression, anxiety or stress with COVID-19 lockdown or after lockdown. The severe anxiety, stress or depression in this population was closely correlated with the reported deterioration in their quality of life which was consistently associated with all outcomes for both women and their partners. This data suggests sex differences in the mental health response to COVID-19, where women remained stressed in lockdown but less anxious than male partners. Regardless of the pandemic mental health was associated with the families’ reported quality of life.

Studies of pregnant populations in North America reported pregnant women were twice as likely to be depressed when pregnant during the pandemic than pre-pandemic [22]. King et al. [22] reported COVID-19 effects related to both objective adversity such as changes to finances, employment and antenatal care and subjective stress such as fear of viral infection or concerns related to support during labour and delivery. Overall, the study found that women with severe stress had higher depressive symptoms with more severe symptoms linked to socioeconomic inequality. These findings are supported by similar studies of depression among pregnant and postpartum women in North America following the onset of the COVID-19 pandemic [23–25]. Guo et al. [6] examined and compared maternal mental health during lockdown across three countries and identified that maternal severe mental health during lockdown was influenced maternal age, quality of life, education, unemployment and socioeconomic status. Our population was predominantly women who were well educated and living in financially stable households which may have buffered any significant impacts of the pandemic on mental health. Those participants that reported a poorer quality of life however were more likely to report severe depression, anxiety and stress regardless of the presence of lockdown or the pandemic. Our findings have uniquely identified that the interaction between mental health and quality of life was also extended to their partners.

There are sex differences in depressive and anxiety related symptoms with women being at greater risk of mental health symptoms than men [26, 27]. A systematic review of 13 studies identified a higher prevalence of anxiety and depression in females than males during the pandemic [28]. During COVID-19 lockdown, Gouvernet and Bonierbale [29] reported French women were more anxious and depressed than men especially women of low socioeconomic groups while lockdown was protective against depression in men. Italian women reported more severe mental health symptoms than men but were more resilient than men during COVID lockdown with improved women reporting mental health outcomes during lockdown [30]. Australian women were more likely than men to have clinically significant symptoms of depression and anxiety due to extra caring responsibilities for children and other dependents, loss of employment, fear of COVID-19 infection and the impact of the restrictions contributing to these changes in mental health [31]. In our cohort we observed a sex difference in relation to a decrease in anxiety for women relative to their partners but depression and stress did not change with lockdown.

The interchange between anxiety and stress during and after lockdown provides an insight into the impact of the pandemic on this population of pregnant women. These outcomes were identified using the DASS 21 [8] which defines anxiety by the assessment of autonomic arousal (dry mouth, hard to breathe), skeletal muscle effects (trembling hands), situational anxiety (social interactions) and subjective experience of anxiety. The stress scale detected sensitivity to levels of chronic non-specific arousal (hard to relax, irritable, intolerant). Anxiety in pregnant women and women with a newborn child in this study were reduced with lockdown possibly due to the removal of situational anxiety that can arise socially and in the workplace. Stress improved after lockdown in both pregnant women and women with a newborn which suggests situations such as home schooling, carer duties, work from home, managing a newborn in isolation and other pressures enforced by the lock down-induced restrictions [31] could have caused irritability and agitation at home which were resolved after lockdown. However, this information was not collected in the current cohort and can only be inferred from the findings of other studies [6, 31].

We identified that maternal depression in this cohort increased with partner depression. It was not identified during the study whether partner depression was paternal perinatal depression or pre-existing depression. The findings support previous data that reported associations between maternal and paternal depression affecting each other in the postpartum period [32]. Conversely, during COVID-19 it was identified that living with a partner during lockdown was beneficial for the depressive symptoms experienced by mothers which may be minimised by social support [29]. Our work identified that relationship satisfaction measured using the CSI was important for reducing maternal depression in the postpartum period in this cohort of families. The current findings indicate depression in both partners may be detrimental in lockdown but can be alleviated to some degree in women if the relationship is supportive. Perinatal care should consider addressing mental health and taking a family centred approach for the management of perinatal mental health during a pandemic.

The present study did have several limitations. Our after lockdown group was only represented by 55 mothers which is likely to have led to underpowered comparisons. This is particularly apparent for depression in mothers at 24 weeks where despite a substantial reduction in the odds of depression in our sample of after lockdown mothers (relative to pre-COVID mothers), the excessively wide confidence intervals suggest we could not infer this association to the population. However, in other cases the small sample size was not enough to mask strong differences among the groups. Most notably, the risk of anxiety at 24 weeks could still be demonstrated to be significantly lowered among post-lockdown, relative to the pre-COVID cohort. Another potential limitation is that while our study accounted for children in the household by parity we did not directly question participants on the number of children and their age who were living within the family home fulltime. This missing data may have limited our understanding of the impact of lockdown on carer responsibilities, home schooling, employment, work from home or fears associated with COVID-19. Greater insight into the impact of these factors on anxiety and stress would have strengthened the findings. Our population was well resourced educationally and financially relative to other populations in Australia which may explain in part why there were no significant changes in mental health outcomes for the partners or for depression symptoms for both members of the families. Overall, the prospective nature of the recruitment was an added strength especially with the ability to compare mothers and partners with families experiencing pregnancy and childbirth before the onset of the pandemic.

A poor quality of life was associated with the development of severe mental health symptoms in this population regardless of the presence of COVID-19. This is an important consideration for directing limited mental health resources towards those more vulnerable populations. For families experiencing a pregnancy or living with a newborn, a family centred approach for mental health clinical care could be advantageous given the influence of paternal depression on maternal mental health especially in the postpartum period. Generally, an educated and financially stable reproductive age population appear to be able to manage the impact of lockdown and the pandemic with some benefits related to reduced anxiety. However, COVID-19 lockdown did not alter stress and strategies to manage stress could be considered in public health communications.

## Supplementary Information

**Additional file 1: Figure S1.** Propensity score adjusted odds for maternal anxiety in relation to each dimension of maternalquality of life at 24 weeks gestation. Overall and individually all quality of life dimensions contribute to maternal anxiety. **Figure S2.** Propensity score adjusted odds for maternal anxiety in relation to each dimension ofmaternal quality of life at 6 weeks postpartum. Overall and individually all quality of life dimensions contribute to maternal anxiety. **Figure S3.** Propensity score adjusted odds for partner anxiety in relation to each dimension of thepartner’s quality of life at 24 weeks gestation. Overall and individually, all quality of life dimensions contribute to partner anxiety. **Figure S4.** Propensity score adjusted odds for maternal depression in relation to each dimension of maternal quality of life at 24weeks gestation. Overall and individually, all quality of life dimensionscontribute to maternal depression except the senses domain. **Figure S5.** Propensity score adjustedodds for maternal depression in relation to each dimension of maternal quality of life at 6 weeks postpartum. Overall and individually, all quality of life dimensions contribute to maternal depression. **Figure S6.** Propensity score adjusted odds for partner depression in relation to each dimension of the partner’s quality of life at 24 weeks gestation. Overall and individually, all quality of life dimensions contribute to partner depression. **Figure S7.** Propensity score adjusted odds for maternal stress in relation to each dimension of maternal quality of life at 24 weeks gestation. Overall and individually, all quality of life dimensions contribute to maternal stress except the senses domain. **Figure S8.** Propensity score adjusted odds for maternal stress in relation to each dimension of maternal quality of life at 6 weeks postpartum. Overall and individually, all quality of life dimensions contribute to maternal stress. **Figure S9.** Propensity score adjusted odds for partner stress inrelation to each dimension of the partner’s quality of life at 24 weeks gestation. Overall and individually, all quality of life dimensions contribute to partner stress. **Figure S10.** Propensity score adjusted odds for maternal postpartum depression in relation to each dimension of maternal quality of life at 6 weeks postpartum. Overall and individually, all quality of life dimensions contribute to maternal postpartum depression.

**Additional file 2: Table S1.** Comparison of complete cases and inclusion of imputed data in maternal dataset. **Table S2.** Comparison of complete cases and inclusion of imputed data in partner dataset. **Table S3a.** Comparison of maternal questionnaire scores between the groups at 24 weeks gestation. **Table S3b.** Comparison of maternal questionnaire scores between the groups at 6 weeks postpartum. **TableS3c.** Comparison of partner questionnaire scores between the groups. **TableS4a. **Propensity score adjusted odds of depression in pregnant women at 24 weeks gestation during and after COVID19 lockdown relative to pregnant women at 24 weeks gestation before the COVID19 pandemic. **Table S4b. **Propensity score adjusted odds of anxiety in pregnant women at 24 weeks gestation during and after COVID19 lockdown relative to pregnant women at 24weeks gestation before the COVID19 pandemic. **Table S4c.** Propensity scoreadjusted odds of stress in pregnant women at 24 weeks gestation during and after COVID19 lockdown relative to pregnant women at 24 weeks gestation before the COVID19 pandemic. **Table S5a. **Propensity score adjusted odds of depression in women at 6 weeks postpartum during and after COVID19 lockdown relative to women at 6 weeks postpartum before the COVID19 pandemic. **Table S5b. **Propensity score adjusted odds of anxiety in women at 6 weeks postpartum during and after COVID19 lockdown relative to women at 6 weeks postpartum before the COVID19 pandemic. **Table S5c. **Propensity score adjusted odds ofstress in women at 6 weeks postpartum during and after COVID19 lockdown relative to women at 6 weeks postpartum before the COVID19 pandemic. **Table S5d. **Propensity score adjusted odds of postpartum depression in women at 6 weeks postpartum during and after COVID19 lockdown relative to women at 6 weeks postpartum before the COVID19 pandemic as measured by EPDS. **Table S6a.** Propensity score adjusted odds of depression in partners at 24 weeks gestation during and after COVID19 lockdown relative to partners at 24 weeks gestation before the COVID19 pandemic. **Table S6b.** Propensity score adjusted odds of anxiety in partners at 24 weeks gestation during and after COVID19 lockdown relative to partners at 24 weeks gestation before the COVID19 pandemic** Table S6c.** Propensity score adjusted odds of stress in partners at 24 weeks gestation during and after COVID19 lockdown relative to partners at 24 weeks gestation before the COVID19 pandemic.

## Data Availability

The datasets generated and/or analysed during the current study are not publicly available due to the ongoing collection and recruitment of participants to the study but are available from the corresponding author following institutional approval.
