# Supplementary material for: Associations between COVID-19 lockdown and post-lockdown on the mental health of pregnant women, postpartum women and their partners from the Queensland family cohort prospective study
Source: BMC Pregnancy Childbirth. 2022 Jun 4;22:468. doi: 10.1186/s12884-022-04795-9 (PMC9166205; doi:10.1186/s12884-022-04795-9)
Supplement: Supplementary file 1 — Additional file 1: Figure S1. Propensity score adjusted odds for maternal anxiety in relation to each dimension of maternalquality of life at 24 weeks gestation. Overall and individually all quality of life dimensions contribute to maternal anxiety. Figure S2. Propensity score adjusted odds for maternal anxiety in relation to each dimension ofmaternal quality of life at 6 weeks postpartum. Overall and individually all quality of life dimensions contribute to maternal anxiety. Figure S3. Propensity score adjusted odds for partner anxiety in relation to each dimension of thepartner’s quality of life at 24 weeks gestation. Overall and individually, all quality of life dimensions contribute to partner anxiety. Figure S4. Propensity score adjusted odds for maternal depression in relation to each dimension of maternal quality of life at 24weeks gestation. Overall and individually, all quality of life dimensionscontribute to maternal depression except the senses domain. Figure S5. Propensity score adjustedodds for maternal depression in relation to each dimension of maternal quality of life at 6 weeks postpartum. Overall and individually, all quality of life dimensions contribute to maternal depression. Figure S6. Propensity score adjusted odds for partner depression in relation to each dimension of the partner’s quality of life at 24 weeks gestation. Overall and individually, all quality of life dimensions contribute to partner depression. Figure S7. Propensity score adjusted odds for maternal stress in relation to each dimension of maternal quality of life at 24 weeks gestation. Overall and individually, all quality of life dimensions contribute to maternal stress except the senses domain. Figure S8. Propensity score adjusted odds for maternal stress in relation to each dimension of maternal quality of life at 6 weeks postpartum. Overall and individually, all quality of life dimensions contribute to maternal stress. Figure S9. Propensity score adjusted odds for partner s [file 12884_2022_4795_MOESM1_ESM.docx]

**Legend for all figures:** AQoL 6D questionnaire domains include **IL:** Independent living **Rel:** Relationships; **MH:** Mental Health; **Cope:** Coping mechanisms; **Pain:** physical pain; **Sen:** senses

**Figure S1** Propensity score adjusted odds for maternal anxiety in relation to each dimension of maternal quality of life at 24 weeks gestation. Overall and individually all quality of life dimensions contribute to maternal anxiety


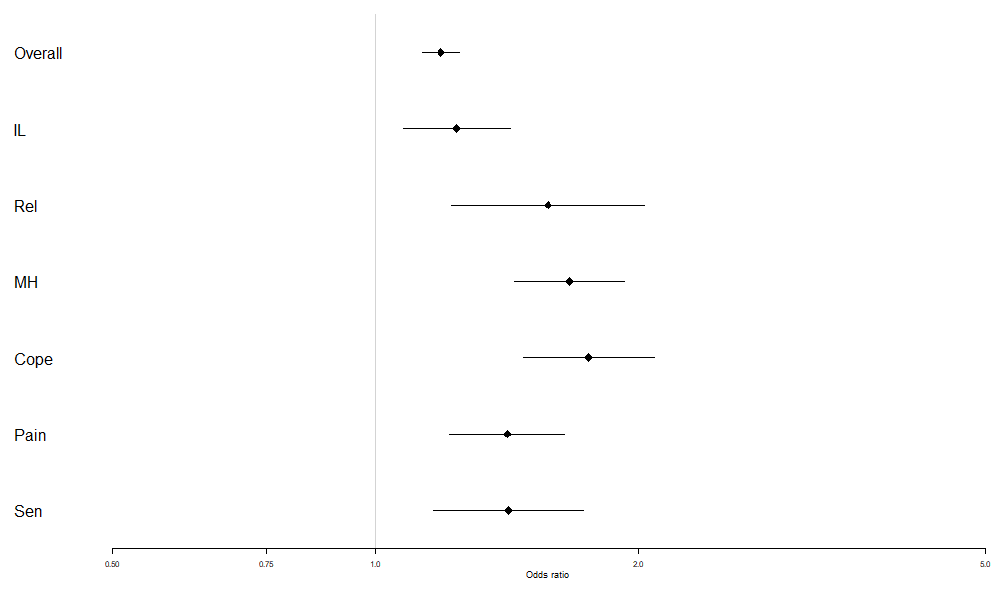


**Figure S2** Propensity score adjusted odds for maternal anxiety in relation to each dimension of maternal quality of life at 6 weeks postpartum. Overall and individually all quality of life dimensions contribute to maternal anxiety


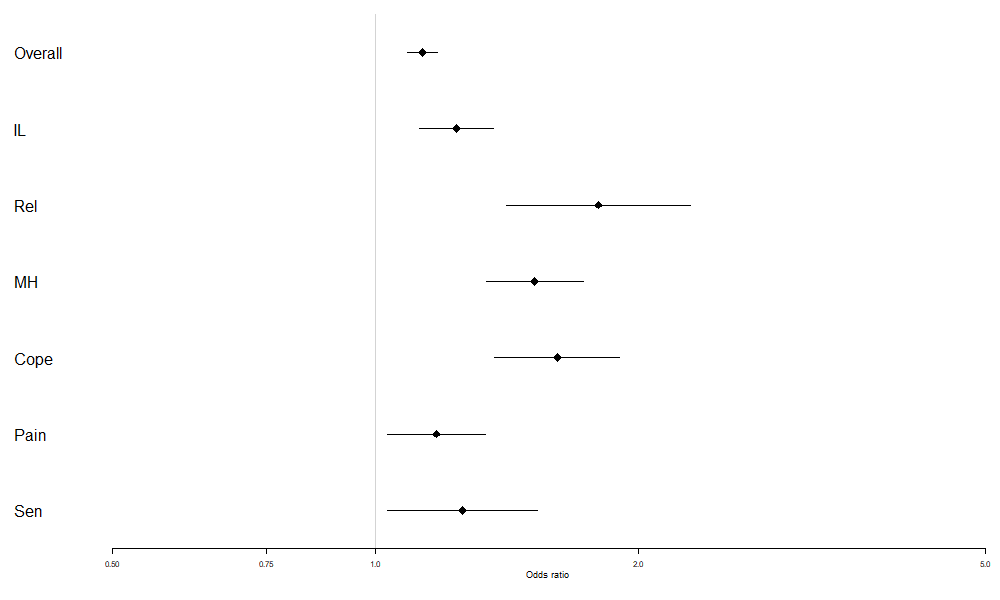


**Figure S3** Propensity score adjusted odds for partner anxiety in relation to each dimension of the partner’s quality of life at 24 weeks gestation. Overall and individually, all quality of life dimensions contribute to partner anxiety


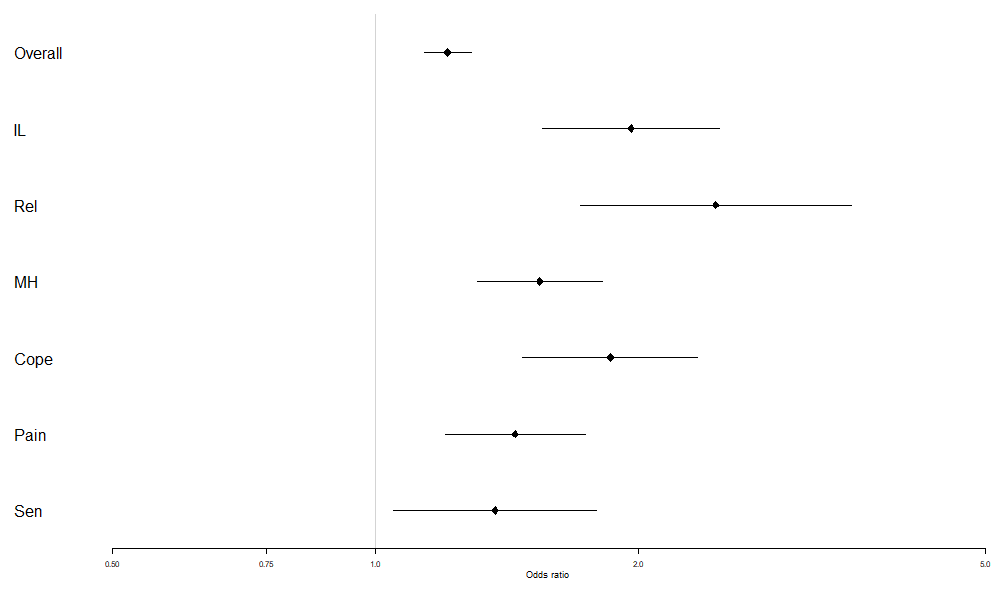


**Figure S4** Propensity score adjusted odds for maternal depression in relation to each dimension of maternal quality of life at 24 weeks gestation. Overall and individually, all quality of life dimensions contribute to maternal depression except the senses domain


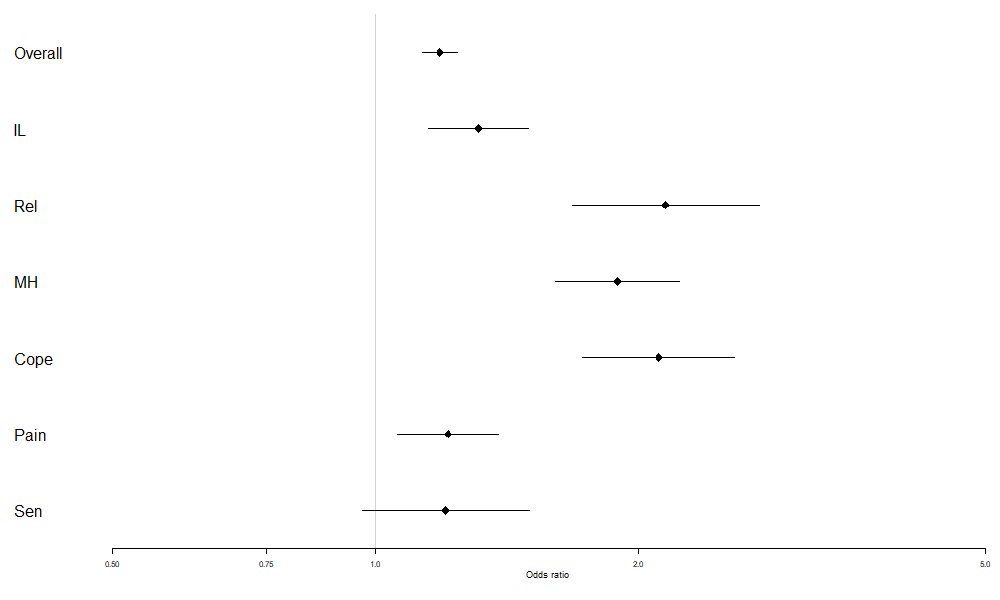


**Figure S5** Propensity score adjusted odds for maternal depression in relation to each dimension of maternal quality of life at 6 weeks postpartum. Overall and individually, all quality of life dimensions contribute to maternal depression

**Figure S6** Propensity score adjusted odds for partner depression in relation to each dimension of the partner’s quality of life at 24 weeks gestation. Overall and individually, all quality of life dimensions contribute to partner depression


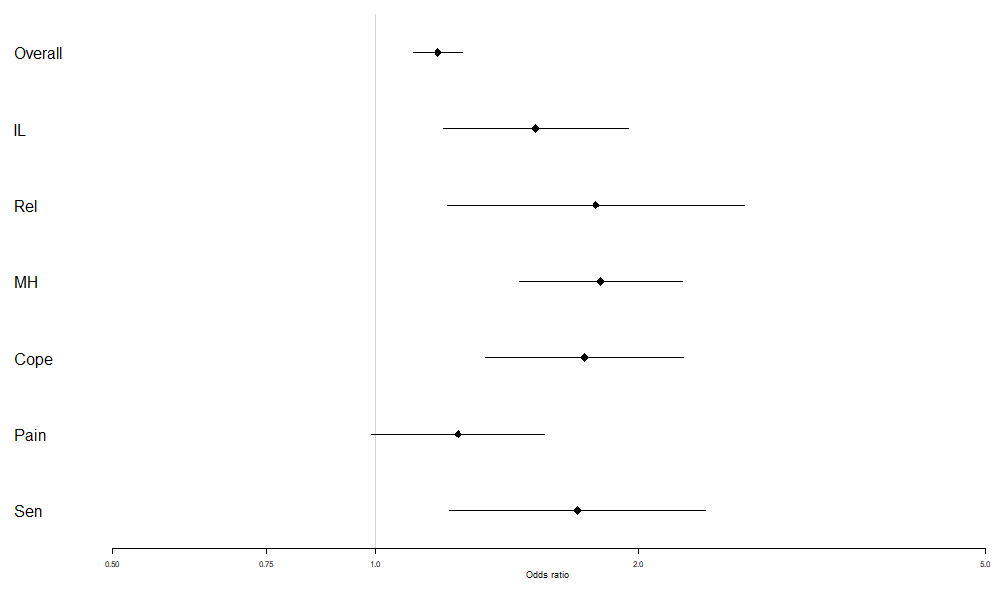


**Figure S7** Propensity score adjusted odds for maternal stress in relation to each dimension of maternal quality of life at 24 weeks gestation. Overall and individually, all quality of life dimensions contribute to maternal stress except the senses domain
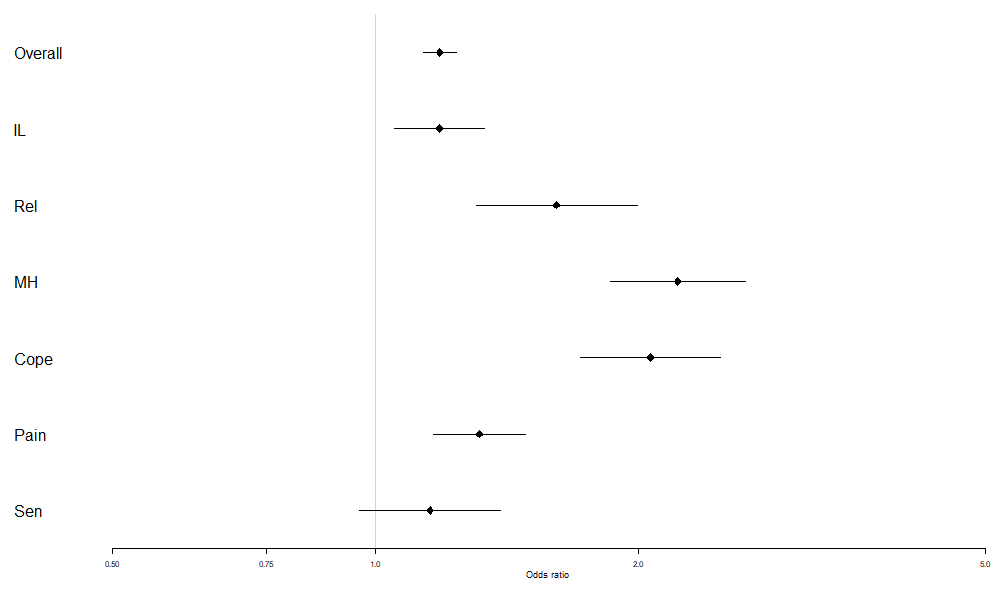


**Figure S8** Propensity score adjusted odds for maternal stress in relation to each dimension of maternal quality of life at 6 weeks postpartum. Overall and individually, all quality of life dimensions contribute to maternal stress


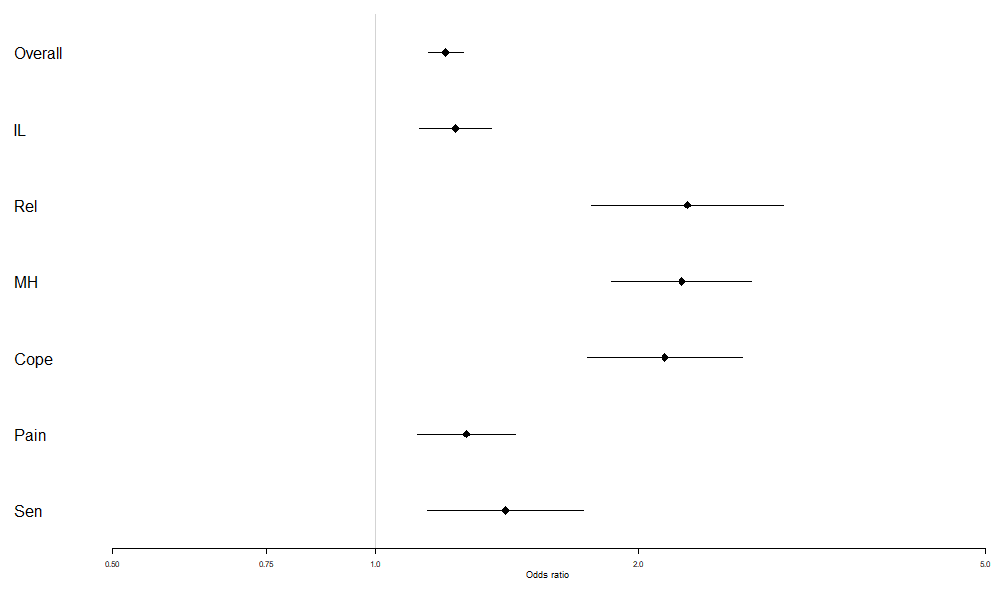


**Figure S9** Propensity score adjusted odds for partner stress in relation to each dimension of the partner’s quality of life at 24 weeks gestation. Overall and individually, all quality of life dimensions contribute to partner stress


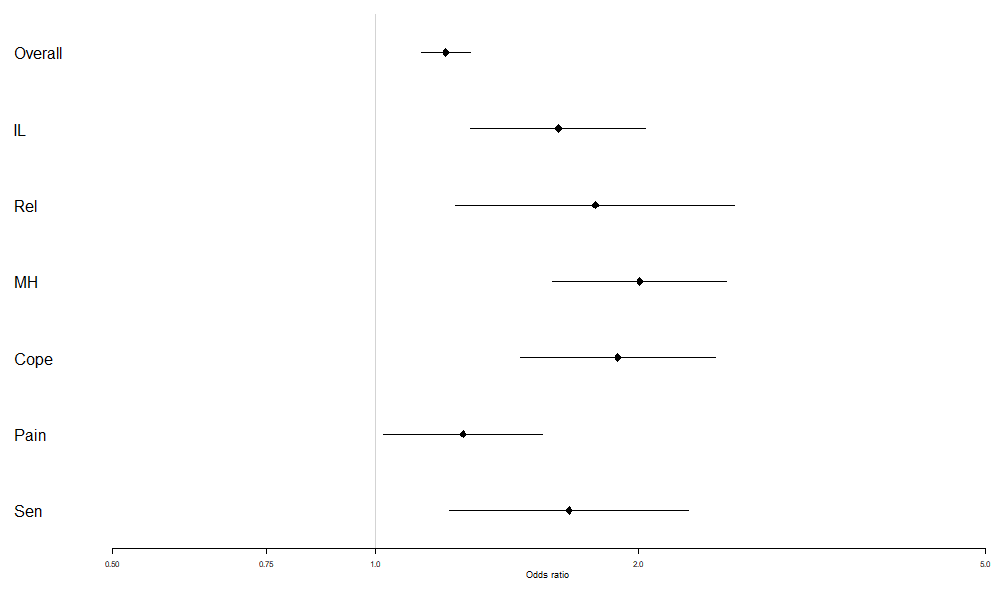


**Figure S10** Propensity score adjusted odds for maternal postpartum depression in relation to each dimension of maternal quality of life at 6 weeks postpartum. Overall and individually, all quality of life dimensions contribute to maternal postpartum depression
