# Supplementary material for: Associations between COVID-19 lockdown and post-lockdown on the mental health of pregnant women, postpartum women and their partners from the Queensland family cohort prospective study
Source: BMC Pregnancy Childbirth. 2022 Jun 4;22:468. doi: 10.1186/s12884-022-04795-9 (PMC9166205; doi:10.1186/s12884-022-04795-9)
Supplement: Supplementary file 2 — Additional file 2: Table S1. Comparison of complete cases and inclusion of imputed data in maternal dataset. Table S2. Comparison of complete cases and inclusion of imputed data in partner dataset. Table S3a. Comparison of maternal questionnaire scores between the groups at 24 weeks gestation. Table S3b. Comparison of maternal questionnaire scores between the groups at 6 weeks postpartum. TableS3c. Comparison of partner questionnaire scores between the groups. TableS4a. Propensity score adjusted odds of depression in pregnant women at 24 weeks gestation during and after COVID19 lockdown relative to pregnant women at 24 weeks gestation before the COVID19 pandemic. Table S4b. Propensity score adjusted odds of anxiety in pregnant women at 24 weeks gestation during and after COVID19 lockdown relative to pregnant women at 24weeks gestation before the COVID19 pandemic. Table S4c. Propensity scoreadjusted odds of stress in pregnant women at 24 weeks gestation during and after COVID19 lockdown relative to pregnant women at 24 weeks gestation before the COVID19 pandemic. Table S5a. Propensity score adjusted odds of depression in women at 6 weeks postpartum during and after COVID19 lockdown relative to women at 6 weeks postpartum before the COVID19 pandemic. Table S5b. Propensity score adjusted odds of anxiety in women at 6 weeks postpartum during and after COVID19 lockdown relative to women at 6 weeks postpartum before the COVID19 pandemic. Table S5c. Propensity score adjusted odds ofstress in women at 6 weeks postpartum during and after COVID19 lockdown relative to women at 6 weeks postpartum before the COVID19 pandemic. Table S5d. Propensity score adjusted odds of postpartum depression in women at 6 weeks postpartum during and after COVID19 lockdown relative to women at 6 weeks postpartum before the COVID19 pandemic as measured by EPDS. Table S6a. Propensity score adjusted odds of depression in partners at 24 weeks gestation during and after COVID19 lockdown relative to pa [file 12884_2022_4795_MOESM2_ESM.docx]

**Supplementary tables**

**Table S1 Comparison of complete cases and inclusion of imputed data in maternal dataset**

**Table S2 Comparison of complete cases and inclusion of imputed data in partner dataset**

**Table S3a Comparison of maternal questionnaire scores between the groups at 24 weeks gestation**

**Legend:** DASS21: Depression, Anxiety and Stress Scale; AQoL: Assessment of Quality of Life 6D; IL: independent living; Rel: relationships; MH: mental health; Cope: Ability to cope; CSI: Couples Satisfaction Index; MSPSS: Multidimensional Scale of Perceived Social Support and SRRS: Social Readjustment Rating Scale. Group 1 is pre-COVID, Group 2 COVID lockdown and Group 3 is post COVID lockdown CI: confidence interval. P significance <0.05

**Table S3b Comparison of maternal questionnaire scores between the groups at 6 weeks postpartum**

**Legend:** DASS21: Depression, Anxiety and Stress Scale; AQoL: Assessment of Quality of Life 6D; IL: independent living; Rel: relationships; MH: mental health; Cope: Ability to cope; CSI: Couples Satisfaction Index; EPDS: Edinburgh Postnatal Depression Scale; MSPSS: Multidimensional Scale of Perceived Social Support and SRRS: Social Readjustment Rating Scale. EPDS: Edinburgh Postnatal Depression Scale; Group 1 is pre-COVID, Group 2 COVID lockdown and Group 3 is post COVID lockdown CI: confidence interval. P significance <0.05

**Table S3c Comparison of partner questionnaire scores between the groups**

**Legend:** DASS21: Depression, Anxiety and Stress Scale; AQoL: Assessment of Quality of Life 6D; IL: independent living; Rel: relationships; MH: mental health; Cope: Ability to cope; CSI: Couples Satisfaction Index; MSPSS: Multidimensional Scale of Perceived Social Support and SRRS: Social Readjustment Rating Scale. Group 1 is pre-COVID, Group 2 COVID lockdown and Group 3 is post COVID lockdown CI: confidence interval. P significance <0.05

**Table S4a** Propensity score adjusted odds of depression in pregnant women at 24 weeks gestation during and after COVID19 lockdown relative to pregnant women at 24 weeks gestation before the COVID19 pandemic

**Legend:** DASS21: Depression, Anxiety and Stress Scale; AQoL: Assessment of Quality of Life 6D; CSI: Couples Satisfaction Index; MSPSS: Multidimensional Scale of Perceived Social Support and SRRS: Social Readjustment Rating Scale. CI: confidence interval. P significance <0.05

**Table S4b** Propensity score adjusted odds of anxiety in pregnant women at 24 weeks gestation during and after COVID19 lockdown relative to pregnant women at 24 weeks gestation before the COVID19 pandemic

**Legend:** DASS21: Depression, Anxiety and Stress Scale; AQoL: Assessment of Quality of Life 6D; CSI: Couples Satisfaction Index; MSPSS: Multidimensional Scale of Perceived Social Support and SRRS: Social Readjustment Rating Scale. CI: confidence interval. P significance <0.05

**Table S4c** Propensity score adjusted odds of stress in pregnant women at 24 weeks gestation during and after COVID19 lockdown relative to pregnant women at 24 weeks gestation before the COVID19 pandemic

**Legend:** DASS21: Depression, Anxiety and Stress Scale; AQoL: Assessment of Quality of Life 6D; CSI: Couples Satisfaction Index; MSPSS: Multidimensional Scale of Perceived Social Support and SRRS: Social Readjustment Rating Scale. CI: confidence interval. P significance <0.05

**Table S5a** Propensity score adjusted odds of depression in women at 6 weeks postpartum during and after COVID19 lockdown relative to women at 6 weeks postpartum before the COVID19 pandemic

**Legend:** DASS21: Depression, Anxiety and Stress Scale; AQoL: Assessment of Quality of Life 6D; CSI: Couples Satisfaction Index; MSPSS: Multidimensional Scale of Perceived Social Support and SRRS: Social Readjustment Rating Scale. CI: confidence interval. P significance <0.05

**Table S5b** Propensity score adjusted odds of anxiety in women at 6 weeks postpartum during and after COVID19 lockdown relative to women at 6 weeks postpartum before the COVID19 pandemic

**Legend:** DASS21: Depression, Anxiety and Stress Scale; AQoL: Assessment of Quality of Life 6D; CSI: Couples Satisfaction Index; MSPSS: Multidimensional Scale of Perceived Social Support and SRRS: Social Readjustment Rating Scale. CI: confidence interval. P significance <0.05

**Table S5c** Propensity score adjusted odds of stress in women at 6 weeks postpartum during and after COVID19 lockdown relative to women at 6 weeks postpartum before the COVID19 pandemic

**Legend:** DASS21: Depression, Anxiety and Stress Scale; AQoL: Assessment of Quality of Life 6D; CSI: Couples Satisfaction Index; MSPSS: Multidimensional Scale of Perceived Social Support and SRRS: Social Readjustment Rating Scale. CI: confidence interval. P significance <0.05

**Table S5d** Propensity score adjusted odds of postpartum depression in women at 6 weeks postpartum during and after COVID19 lockdown relative to women at 6 weeks postpartum before the COVID19 pandemic as measured by EPDS

**Legend:** EPDS: Edinburgh Postnatal Depression Scale; DASS21: Depression, Anxiety and Stress Scale; AQoL: Assessment of Quality of Life 6D; CSI: Couples Satisfaction Index; MSPSS: Multidimensional Scale of Perceived Social Support and SRRS: Social Readjustment Rating Scale. CI: confidence interval. P significance <0.05

**Table S6a** Propensity score adjusted odds of depression in partners at 24 weeks gestation during and after COVID19 lockdown relative to partners at 24 weeks gestation before the COVID19 pandemic

**Legend:** DASS21: Depression, Anxiety and Stress Scale; AQoL: Assessment of Quality of Life 6D; CSI: Couples Satisfaction Index; MSPSS: Multidimensional Scale of Perceived Social Support and SRRS: Social Readjustment Rating Scale. CI: confidence interval. P significance <0.05

**Table S6b** Propensity score adjusted odds of anxiety in partners at 24 weeks gestation during and after COVID19 lockdown relative to partners at 24 weeks gestation before the COVID19 pandemic

**Legend:** DASS21: Depression, Anxiety and Stress Scale; AQoL: Assessment of Quality of Life 6D; CSI: Couples Satisfaction Index; MSPSS: Multidimensional Scale of Perceived Social Support and SRRS: Social Readjustment Rating Scale. CI: confidence interval. P significance <0.05

**Table S6c** Propensity score adjusted odds of stress in partners at 24 weeks gestation during and after COVID19 lockdown relative to partners at 24 weeks gestation before the COVID19 pandemic

**Legend:** DASS21: Depression, Anxiety and Stress Scale; AQoL: Assessment of Quality of Life 6D; CSI: Couples Satisfaction Index; MSPSS: Multidimensional Scale of Perceived Social Support and SRRS: Social Readjustment Rating Scale. CI: confidence interval. P significance <0.05
